# One-time relieving of frozen shoulder motor dysfunction with pure acupotomy: A case report

**DOI:** 10.1097/MD.0000000000036783

**Published:** 2023-12-29

**Authors:** Kangyan Zhou, Xiaolin Xie, Jie Liu, Jing Tao, Qiong Liu, Nan Zhou, Wenchao Zhou, Yinli Tao, Yongliang Chen

**Affiliations:** a Chengdu University of Traditional Chinese Medicine, Sichuan, China; b Zhongxian Hospital of Traditional Chinese Medicine, Chongqing, China.

**Keywords:** case report, frozen shoulder, pure acupotomy

## Abstract

**Background:**

Frozen shoulder (FS) is characterized by shoulder pain and restricted movement of the shoulder joint. While it tends to resolve on its own, it significantly affects an individual quality of daily life. The pure acupotomy technique employs needle-knife manipulation as the sole treatment, without the use of medications, such as steroids or vitamins, and local anesthesia if necessary. It aims to restore soft tissue mechanical balance and circulation through techniques such as cutting and stripping, creating a “gap effect.” This technique can rapidly, safely, and effectively relieve functional impairments in patients with FS. This article presents a case study of the successful treatment of FS using a purely needle-knife technique.

**Patient concerns:**

The patient, aged 57 years, presented with chronic pain in the right shoulder, which was particularly aggravated at night, and moderate limitations in joint mobility.

**Diagnoses:**

The patient was diagnosed with periarthritis of the right shoulder (moderate FS, frozen period), type 2 diabetes, and supraspinatus tendinitis of the right shoulder.

**Interventions:**

Conventional treatments, such as topical analgesics and acupuncture, produced insignificant improvements in symptoms. So, the patient chose acupotomy treatment and signed the treatment consent form.

**Outcomes:**

After undergoing one minimally invasive acupotomy treatment, the patient experienced immediate restoration of normal shoulder joint mobility and a significant reduction in pain intensity 3 days post-treatment.

**Lessons:**

We believe that utilizing a purely acupotomy treatment for passive functional impairments in FS not only yields good results but also saves patients time and reduces their financial burden. This is worth promoting extensively in clinical practice.

## 1. Introduction

Frozen shoulder (FS), characterized by stiffness of the shoulder joint, is a condition often described as a “disease that is difficult to define, difficult to treat, and difficult to explain pathologically.”^[1]^ It manifests primarily as pain and restricted joint movement and can be categorized into primary and secondary forms, commonly resulting from trauma, overuse, or degenerative processes. The disease course is protracted and can even linger, severely affecting the daily lives of affected individuals.^[2]^ Current treatment options include oral non-steroidal anti-inflammatory drugs, intraarticular injections of steroids and local anesthetics, acupuncture, physical therapy, and manual release techniques following nerve block anesthesia. However, there is a limited body of targeted literature focusing on the rapid restoration of passive functional movements, such as contralateral scapula palpation during shoulder joint extension and flexion, abduction, or anterior elevation, a challenge commonly encountered in clinical practice.

Acupotomy therapy represents a novel, minimally invasive treatment approach that combines traditional Chinese acupuncture with modern surgical techniques. It has a short course of treatment and quick results and has gradually become one of the main treatments for FS.^[3]^ Pure acupotomy therapy specifically refers to the use of a needle-knife without the use of corticosteroids, vitamins, or other medications, with local anesthesia administered when necessary. This technique involves precise needle insertion, manipulation for tonifying or reducing, and meticulous anatomical understanding to achieve therapeutic goals.^[4]^ This technology can one-time relieve FS dysfunction, so that motor functions such as extending and flexing the elbow to touch the opposite scapula, lifting in abduction or forward flexion can return to normal.

In this paper, we present a case demonstrating the application of pure acupotomy as a one-time intervention for relieving dysfunction in a patient with FSs.

## 2. Case presentation

**Figure 1.**
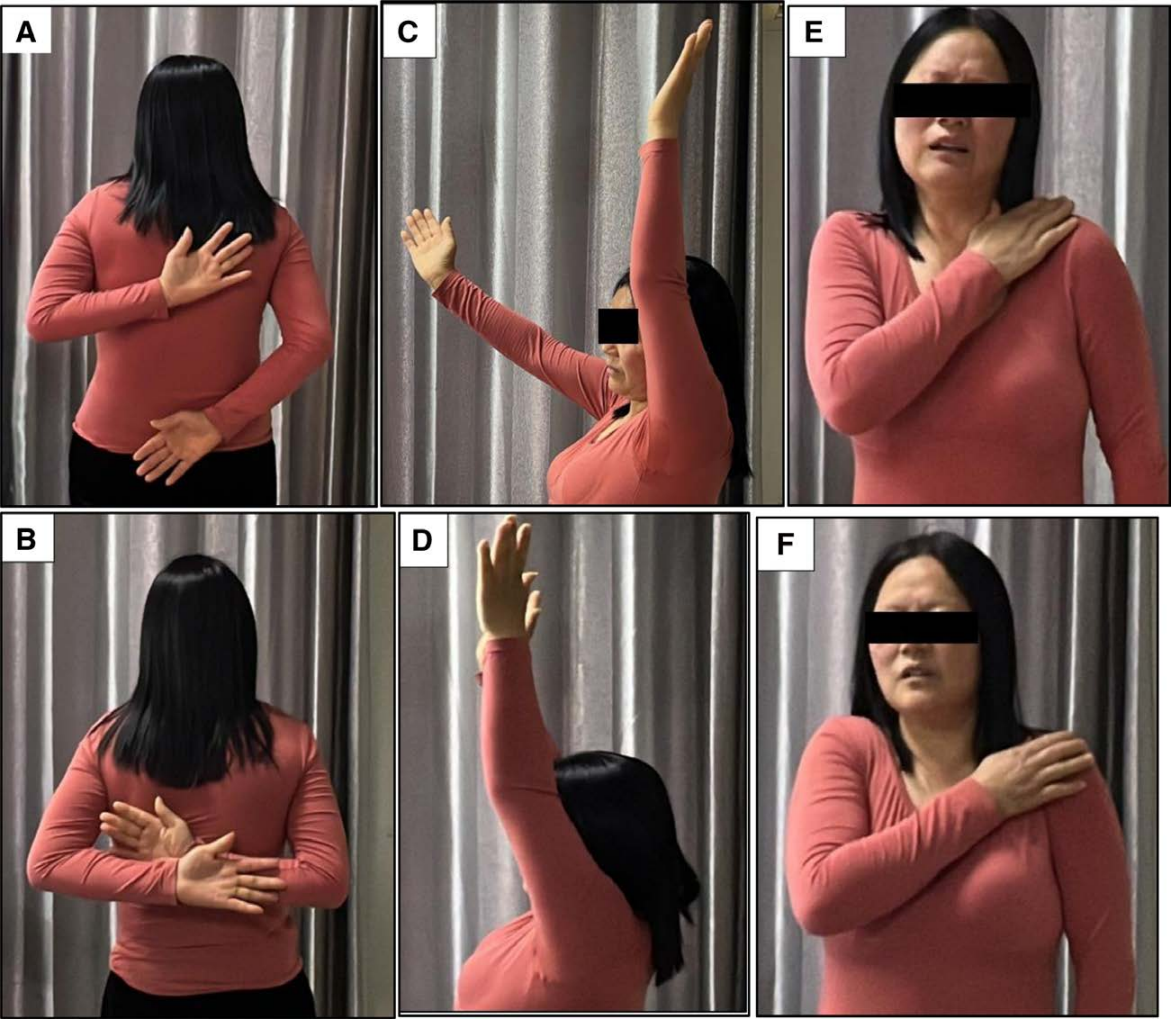
Shows a comparison of shoulder joint motion function before and after a single pure acupotomy treatment in a patient with frozen shoulder. (A, B) Comparison of shoulder joint backward extension and elbow flexion touching the contralateral scapula before and after treatment. (C, D) Comparison of shoulder joint forward flexion and lifting before and after treatment. (E, F) Comparison of shoulder joint internal rotation and elbow adduction touching the contralateral shoulder before and after treatment).

This is the case of a 57-year-old female patient who presented with right shoulder pain and limited joint mobility for > 3 months. The lifting range of motion of the shoulder joint was approximately 110°in the forward flexion position and approximately 115° in the abduction position, and the thumb was able to reach the ipsilateral buttock during extension. Palpation revealed moderately tender points in the infraspinatus, teres minor, deltoid, and clavicular portions of the pectoralis major muscle. The visual analog scale (VAS) score was 3. Magnetic resonance imaging of the right shoulder joint showed adhesive capsulitis of the right shoulder joint, degenerative tendinitis of the right supraspinatus muscle, small cysts under the articular surface of the right humeral head, and mild fluid accumulation around the subacromial-subdeltoid bursa, subscapularis bursa, and tendon sheath of the long head of the biceps brachii. After ruling out other conditions through relevant laboratory tests and examinations, the patient was diagnosed with “periarthritis of the right shoulder (moderate FS,^[5]^ frozen period^[6]^), type 2 diabetes, and supraspinatus tendinitis of the right shoulder.” Traditional acupuncture therapy was initially performed according to the patient preference. One week later, the patient reported no relief from pain. Widespread tenderness and multiple trigger points were palpated around the right shoulder and the scapular region. The VAS score remained at 3, and the range of motion in the right shoulder joint improved slightly, with approximately 120° in the forward flexion position, 120° in the abduction position, the thumb reaching the ipsilateral buttock during extension, and the middle finger of the right hand can’t touch the opposite shoulder peak (Figure 1A,C,E). After excluding contraindications such as severe anemia, coagulation disorders, and significant organ dysfunction, and following thorough communication and obtaining informed consent, the patient agreed to undergo acupotomy therapy.

The operational procedures were as follows: the treatment room was pre-disinfected with ultraviolet light, and disposable bed sheets were used. The patient was initially positioned in a prone position with the right upper limb abducted at 90°. Subsequently, the patient was placed in a supine position with the right upper limb abducted at 45°and the palm facing upward to adequately expose the treatment area. The acupotomy treatment points were identified through palpation, focusing on tender or positive reaction points, and were marked with a marker pen (Table 1 for specific points). The operator wore a disposable medical cap, surgical mask, surgical gown, and gloves. A drape was placed over the affected area and the local skin was disinfected with iodine. Based on the marked points, a 4-step protocol was followed for needle-knife insertion. The incision line was maintained parallel to the important nerves, blood vessels, and muscle fibers, and the needle-knife was inserted slowly until it reached the bone surface (note: caution should be exercised to avoid deep penetration into the thoracic cavity). Subsequently, the angle of the incision line was adjusted, or cutting and peeling were performed directly, approximately 3 to 5 incisions per location, stopping depending on looseness.

**Table 1:** Selected points for acupotomy of the right shoulder joint.

Position	Shoulder region	Treatment points
Prone position	Posterior shoulder Scapular region	Origin and insertion points and tendon nodes of the Teres minor muscle Tenderness points of the infraspinatus muscle
Supine position	Anterior shoulder The anterior aspect of the upper arm	Tendon nodes at the starting point of pectoralis major muscle Coracoid process Lesser tubercle of the humerus Intertubercular groove Lesser/greater tuberosity crest of the humerus

Following completion of the acupotomy, manual stripping was immediately performed: lifting at the abduction position: the patient was in a supine position, with the right side close to the edge of the bed. One assistant stood on the left side of the patient and stabilized the ribs on the affected side, while the other assistant exerted force on the shoulder well region from the head side to the foot side. The operator stood on the patient right side, holding the upper 1/3 of the humerus of the affected limb with both hands. The patient was instructed to “cough” and actively initiate abduction until the shoulder joint reached its limits. At that moment, the operator swiftly assisted the patient in achieving maximal abduction to the functional position, accompanied by a crisp “creaking” sound, indicating the release of adhesions. Stretching and bending the elbow to touch the lower angle of the opposite scapula: The patient sat in a chair, with 1 assistant providing support to stabilize her position. The operator held the patient shoulder with the right hand and supported the middle shaft of the forearm with the left hand, exerting gentle pressure. The patient arm was repeatedly stretched and bent the elbow with the affected limb and tried to touch the inferior angle of the contralateral scapula. During this process, the operator engaged the patient in a conversation to distract her attention. When the patient arm reached the limit of extension, the operator gently lifted the arm to the maximum functional position, accompanied by a crisp “creaking” sound, indicating the release of adhesions. Adduction with elbow flexion against the shoulder: the patient was in a sitting position, with 1 assistant providing support to stabilize the patient trunk. The patient was instructed to flex the right elbow towards the chest, allowing the inner side of the elbow joint to touch the chest. The operator held the patient shoulder joint with one hand and supported the posterior aspect of the patient elbow with the other hand, applying no force. The patient was asked to actively use the middle finger of the right hand to repeatedly touch the anterior aspect of the contralateral shoulder joint. The operator engaged the patient in a conversation to distract her attention. When the movement reached the limit, the operator gently assisted in achieving a slight movement, accompanied by a “creaking” sound, indicating the release of adhesions.

The entire treatment procedure lasted approximately 15 minutes. Immediately after the treatment, the patient right shoulder joint mobility was assessed: lifting 180° in forward flexion position and 180° in abduction position, and during backward extension and flexion of the elbow, the thumb reached the level of the 6th-7th ribs on the chest, and the middle finger of the right hand can touch the opposite shoulder peak (Figure 1B,D,F). Additionally, the patient was instructed not to expose the incision to water within the next 3 days to prevent infection. The patient was advised to engage in moderate shoulder joint activities, and avoid lifting heavy objects and doing violent stretching to prevent further injury. After 3 days, the VAS score was 2. A follow-up assessment at 3 months revealed that the patient had not received any additional treatment during this period, and the shoulder joint regained normal mobility without significant pain.

## 3. Discussion

The study of FS in modern medicine has a history of nearly 150 years. In 1872, Duplay first proposed the concept of shoulder periarthritis, which refers to shoulder pain and limited mobility caused by soft tissue lesions around the shoulder joint.^[7]^ In 1934, Codman introduced the concept of shoulder stiffness and designated it as FS, distinguishing it from shoulder periarthritis.^[8]^ In 1945, based on anatomical dissection of cadavers and clinical observations, Neviaser named it adhesive capsulitis.^[9]^ Currently, there is no universally recognized best treatment for FS. For most patients, conservative treatment is the preferred option. This includes intra-articular steroid injections, commonly used in clinical practice and providing rapid symptom relief. However, long-term use of steroids can lead to brittle changes in related tendons and ligaments, and even rupture, making it contraindicated in patients with diabetes. Surgical intervention is only considered in approximately 5% of patients who do not respond to non-surgical treatments, but there is significant controversy surrounding its efficacy.^[10]^

The etiology of FS remains unclear, but it is generally believed to be the result of a combination of synovitis and capsular fibrosis.^[11]^ Cellular factors such as tumor necrosis factor-alpha, transforming growth factor, and platelet-derived growth factor may be involved in this process.^[12]^ Epidemiological studies have confirmed the association between FSs and diabetes, and there is a statistically significant difference in the age and duration of diabetes between individuals with and without FSs.^[13]^ Additionally, other factors such as thyroid diseases, cardiovascular diseases (e.g., myocardial infarction and pulmonary tuberculosis), tumor diseases, neurological disorders (e.g., stroke), medications (e.g., protease inhibitors), smoking, and Dupuytren contracture can also trigger FS.^[14]^ Based on the theory of human bowstring anatomy and netting theory, the coracoid process plays a crucial role in the pathogenesis of a FS. A previous study^[15]^ suggested that abnormal stress on the short head of the biceps brachii at the coracoid process is the primary cause of shoulder diseases. Subsequently, compensatory mechanisms involving adhesion, scar formation, and contracture come into play according to the principles of the human bowstring anatomy, resulting in a 3-dimensional network-like pathological framework. Based on shoulder joint movement-related muscles (Table 2 for specific points) and long-term clinical observation, our team has observed that hidden lesions of the pectoralis minor manifested as long-term muscle atrophy caused by prolonged hunching, are also significant factors contributing to shoulder diseases. Abnormal stress at the coracoid process can alter scapular positioning and affect the movement trajectory of shoulder muscles, such as the subscapularis muscle. This abnormal mechanical transmission by the subscapularis muscle further activates compensation in the rotator cuff tissues (supraspinatus, infraspinatus, teres minor, and subscapularis), and may even cause lesions in neighboring muscles such as the latissimus dorsi and teres major, leading to widespread shoulder and back pain or restricted shoulder joint mobility. Therefore, the mechanical imbalance following soft tissue injuries in the shoulder is an important pathological mechanism of FS, and restoring mechanical balance is a key aspect of its treatment.

**Table 2:** Muscles related to shoulder joint motion.

Shoulder joint movement directions	Main associated muscles
Flexion	Anterior deltoid Clavicle of pectoralis major Coracobrachialis The long head of the biceps brachii
Back extension	Posterior deltoid Latissimus dorsi Teres major The long head of triceps brachii The sternal portion of the pectoralis major
Abduction	Deltoid muscle Supraspinatus muscle Pectoralis major muscle
Adduction	Pectoralis major muscle Latissimus dorsi muscle Teres major muscle Subscapularis
External Rotation	Posterior deltoid muscle Infraspinatus muscle Teres minor muscle
Internal Rotation	Anterior deltoid muscle Pectoralis major muscle Subscapularis Teres major muscle Latissimus dorsi muscle

The needle-knife technique is a combination of surgical knives and acupuncture needles. It not only relieves local lesions, improves mechanical balance, and restores blood supply through the cutting and separation actions of surgical knives but also utilizes the therapeutic effects of acupuncture, such as promoting meridian circulation, activating blood flow, and alleviating pain. Guided by precise anatomy and network theories, the application of the needle-knife technique varies according to the individual, the specific disease, and the pathological structure.^[16]^ A meta-analysis^[17]^ has shown that the efficacy of the needle-knife technique is significantly superior to traditional acupuncture and electroacupuncture in the treatment of shoulder periarthritis.

The patient, 57 years old, according to traditional Chinese medicine theory, is in the phase where the Ren meridian and the Tai Chong meridian gradually become deficient after reaching the age of forty-nine in women. The essence and qi of the 5 Zang organs and 6 Fu organs gradually decline, resulting in insufficient qi and blood to properly nourish the tendons, muscles, and joints. Additionally, the patient long-term heavy physical work, combined with the prevalence of cold and dampness during the rainy season, contributed to the onset of the condition, as described in the “Su Wen, Bi Lun,”^[18]^ which states, “When wind, cold, and dampness combine, it becomes bi (obstruction) disease.” The main symptoms of the patient included pain in the right shoulder joint and significant restriction of movement in multiple directions, severely affecting daily life. After more than a week of acupuncture treatment with unsatisfactory improvement, a decision was made to proceed with the acupotomy technique. On thorough physical examination, the patient was found to have widespread moderate tenderness in the infraspinatus, teres minor, teres major, deltoid, and pectoralis major muscles on the right side. Additionally, palpable fibrous adhesions of varying degrees were detected in multiple muscles surrounding the right shoulder. Combined with relevant imaging examinations, other diseases were ruled out, and a diagnosis of FS was established, confirming the use of the acupotomy technique. The treatment approach involved primarily cutting and dispersing the fibrous adhesions, puncturing and reducing fluid accumulation when necessary, and performing moderate cutting and dissecting techniques on the muscles with abnormal mechanical tension. This aimed to create favorable conditions for the separation of adhesions and further promote the functional recovery of the affected shoulder joint by utilizing the gapping effect and facilitating the release of tension in the soft tissues.

This is a common clinical condition for which various treatment methods are available. However, the acupotomy technique offers the advantage of resolving passive movement restrictions of the shoulder joint in a single session, thus providing a fast, safe, and effective approach. This significantly reduces the economic and time costs for patients and brings good news to a large number of FS patients. However, this technique requires a high level of clinical experience and a solid foundation of professional knowledge from the operator. It necessitates systematic training for widespread adoption and requires ongoing efforts from dedicated teams and professionals.

## 4. Conclusion

In conclusion, pure needle-knife treatment for functional impairment in FS offers a green, fast, safe, and effective approach. Importantly, it significantly reduces patients’ time and economic costs, bringing good news to a wide range of clinical patients.

## Author contributions

**Conceptualization:** Kangyan Zhou, Yongliang Chen.

**Data curation:** Qiong Liu, Nan Zhou.

**Formal analysis:** Kangyan Zhou.

**Investigation:** Xiaolin Xie.

**Methodology:** Xiaolin Xie, Jie Liu, Jing Tao, Wenchao Zhou, Yinli Tao, Yongliang Chen.

**Project administration:** Yongliang Chen.

**Supervision:** Jing Tao, Nan Zhou.

**Writing – original draft:** Kangyan Zhou.

**Writing – review & editing:** Kangyan Zhou, Yongliang Chen.

## References

[1] Jun L Chen W. Advances in diagnosis and treatment of frozen shoulder. Chin J Joint Surg (Electronic Edition) 2015;9:527–31.

[2] Zhenfei L. CTA in vivo anatomical measurement and clinical research of frozen shoulder [D]. Nanjing University of Traditional Chinese Medicine, 2020. DOI: 10.27253/d.cnki.gnjzu.2020.000235.

[3] Xiangyu J Shengyong S Xiaozhen H. Research progress on the mechanism of acupotomy treatment of frozen shoulder. J Liaoning Univ Tradit Chin Med. 2019;21:162–4.

[4] Yongliang C. Clinical Research on Zhongzhou Pure Acupotomy Treatment of the Frozen Shoulder. Chongqing, Zhongxian Hospital of Traditional Chinese Medicine, 2021.

[5] Edited by Garsman Gary M., translated by Xu Weidong, Chen Shiyi, and Chen Baicheng. Shoulder Arthroscopy [M]. Shanghai: Second Military Medical University Press, 2008:139.

[6] Le HV Lee SJ Nazarian A. Adhesive capsulitis of the shoulder: a review of pathophysiology and current clinical treatments. Shoulder Elbow 2017;9:75–84. 28405218 10.1177/1758573216676786 PMC5384535

[7] Yue D Junchao T Guoshuai Y. Research progress of frozen shoulder. Clin Med Progress 2023;13:7761–4.

[8] Codman EA. The Shoulder: Rupture of the Supraspinatus Tendon and other Lesions in or About the Subacromial Bursa. Boston: Thomas Todd Co,1934.

[9] Nevaiser TJ. Adhesive capsulitis of the shoulder: a study of the pathological findings in periarthritis of the shoulder. J Bone Joint Surg. 1945;27:211–22.

[10] Yanhua W Jianhai C. Consolidating the shoulder: the expert consensus of the ISAKOS Upper Limb Committee (Part 2). Chin Elect J Shoulder Elbow Surg. 2017;5:61–5.

[11] Qiuke W Li Z Chunxi Y. Research progress on the mechanism of inflammation and fibrosis in the frozen shoulder. Chin J Joint Surg (Electronic Edition) 2017;11:289–92.

[12] Yanfu T Shuangchun A. Research progress on the pathogenesis of frozen shoulder. Chin J Gerontol. 2020;40:5371–5.

[13] Tighe CB Oakley WS Jr. The prevalence of a diabetic condition and adhesive capsulitis of the shoulder. South Med J. 2008;101:591–5. 18475240 10.1097/SMJ.0b013e3181705d39

[14] D’Orsi GM Via AG Frizziero A. Treatment of adhesive capsulitis: a review. Muscles Ligaments Tendons J. 2012;2:70–8. 23738277 PMC3666515

[15] Qiang Z Tianmin Z. The analysis of the three-dimensional network-like pathological framework of periarthritis of the shoulder by acupotomy medicine. Clin J Acupuncture Moxibustion 2016;32:84–6.

[16] Hanzhang Z. Principles of Acupotomy Medicine [M]. Beijing: People’s Medical Publishing House, 2002

[17] Fushui L Xiaofei J Changqing G. A systematic review of the efficacy of acupuncture and acupotomy in treating periarthritis of the shoulder. Zhonghua J Tradit Chin Med. 2012;27:582–5.

[18] Huangdi Neijing [M]. Beijing: People’s Health Publishing House, 2006.

